# The Board of Dental Examiners

**Published:** 1898-11

**Authors:** G. W. Klump


### The Board of Dental Examiners.

The Board of Dental Examiners of the State of Pennsylvania organized in Harrisburg October 1st by electing Dr. Henry Gerhart, of Lewisbnrg, President, and Dr. G. W. Klump, of Williamsport, Secretary, for the ensuing year.

The next examinations of applicants for license to practice Dentistry in Pennsylvania will commence December 6th, simultaneously in Philadelphia and Pittsburg.

Applications to the Dental Council, Harrisburg, for permission to come before the Board should state in which city the applicant desires to be examined. G. W. KLUMP, *Sec'y.*

